# EFFECTS OF LONG-TERM ROUX-EN-Y GASTRIC BYPASS ON BODY WEIGHT AND CLINICAL METABOLIC COMORBIDITIES IN BARIATRIC SURGERY SERVICE OF A UNIVERSITY HOSPITAL

**DOI:** 10.1590/0102-6720201600S10006

**Published:** 2016

**Authors:** Cátia Ferreira da SILVA, Larissa COHEN, Luciana d'Abreu SARMENTO, Felipe Monnerat Marino ROSA, Eliane Lopes ROSADO, João Régis Ivar CARNEIRO, Antônio Augusto Peixoto de SOUZA, Fernanda Cristina Carvalho Mattos MAGNO

**Affiliations:** 1 Bariatric Surgery Program, Clementino Fraga Filho Hospital, Federal University of Rio de Janeiro; 2 Josué de Castro Nutrition Institute, Federal University of Rio de Janeiro, Rio de Janeiro, RJ, Brazil

**Keywords:** Anastomosis, Roux-en-Y, Diabetes Mellitus, Type 2, Hypertension, Dyslipidemia

## Abstract

**Background:**

Due to the high failure rate observed in the clinical treatment of morbid obesity an increase in bariatric surgery indications, as an alternative for the control of obesity and comorbidities, is noticeable.

**Aim:**

To evaluate the performance of type 2 diabetes mellitus, high blood pressure and dyslipidemia in patients submitted to Roux-en-Y gastric bypass in late follow-up.

**Methods:**

Retrospective analysis of 59 patients included in the bariatric surgery program. Anthropometric (height and body weight) and laboratory (LDLc, HDLc, VLDLc, triglyceride -TG - and glucose) data were collected on pre- and postoperative stages, through medical records.

**Results:**

Among the patients, 86% were female aged 43±11, of whom 52% had attended high school. The average postoperative time was 7±3 years. During the postoperative period, there were decreases of weight and body mass index, respectively (133±06 kg vs 91±04 kg p<0.05 e 49±74 kg/m^2^ vs 33±79 kg/m^2^, p<0.05). In comparison to the preoperative stage, lower concentrations of glucose (101.00±26.99 vs 89,11±15.19, p=0.014), total cholesterol rates (179.00±37,95 vs 167.48±28,50, p=0.016), LDLc (104.30±33.12 vs 91.46±24.58, p=0.016), VLDLc (25.40±11,12 vs 15.68±7.40, p<0.01), and TG (143.35±86.35 vs 82.45±37.39, p<0.01) and higher concentrations of HDLc (43.53±8.23 vs 57.90±15.60, p<0.01) were identified in the postoperative stage. 40% of hypertensive patients were still undergoing high blood pressure treatment during the postoperative stage. There was remission of type 2 diabetes mellitus and dyslipidemia on 81% and 94% of the cases, respectively.

**Conclusion:**

Roux-en-Y gastric bypass has proven itself to be an effective long term procedure, promoting weight loss, remission of DM2 and dyslipidemia.

## RESUMO

**Racional:**

Em função do alto grau de falência que se observa no tratamento clínico da obesidade mórbida, observa-se um aumento da procura pela cirurgia bariátrica como alternativa para o controle da obesidade e comorbidades.

**Objetivo:**

Avaliar a evolução do diabete melito tipo 2, da hipertensão arterial sistêmica e da dislipidemia em pacientes submetidos à gastroplastia redutora em Y-de-Roux no período de pós-operatório tardio.

**Métodos:**

Análise retrospectiva de 59 pacientes inseridos em programa de cirurgia bariátrica. Foram coletados dados antropométricos (altura e peso corporal) e laboratoriais (LDLc, HDLc, VLDLc, triglicerídeo -TG - e glicose) nos períodos pré e pós-operatório por meio de prontuários médicos.

**Resultados:**

Entre os pacientes, 86% eram mulheres com idade de 43±11 anos e 52% tinham cursado o ensino médio. O tempo médio de pós-operatório foi de 7±3 anos. Houve redução no peso e no índice de massa corporal no pós-operatório, respectivamente (133±06 kg vs 91±04 kg p<0,05 e 49±74 kg/m^2^ vs 33±79 kg/m^2^, p<0,05). Observou-se concentrações inferiores no pós-operatório, comparado com o pré-operatório, da glicose (101,00±26,99 vs 89,11±15,19, p=0,014), colesterol total (179,00±37,95 vs 167,48±28,50, p=0,016), LDLc (104,30±33,12 vs 91,46±24,58, p=0,016), VLDLc (25,40±11,12 vs 15,68±7,40, p<0,01), e TG (143,35±86,35 vs 82,45±37,39, p<0,01) e maiores de HDLc (43,53±8,23 vs 57,90±15,60, p<0,01 ). No pós-operatório 40% dos pacientes hipertensos ainda estavam em tratamento para hipertensão arterial sistêmica. Houve remissão do diabete melito tipo 2 e da dislipidemia em 81% e 94% dos casos, respectivamente.

**Conclusão:**

A gastroplastia redutora em Y-de-Roux mostrou ser procedimento eficaz em longo prazo, com resultados persistentes na perda de peso, remissão do DM2 e da dislipidemia.

## INTRODUCTION

The World Health Organization (WHO) considers obesity one of the most serious public health issues of the modern world. Due to the increase of its occurrence and to the severe consequences overweight might lead to, obesity is today considered the most important nutritional disorder in developed and developing countries^30^.

Severe obesity patients are more likely to develop type 2 Diabetes Mellitus (DM2), high blood pressure (HBP) and dyslipidemia, among other diseases. To these individuals, as to those with body mass index (BMI) equal or lower than 35 kg/m² and that already have associated diseases, bariatric surgery is already being considered an important therapeutic alternative^9^ ^,^ ^20^.

Among the techniques used for this procedure, Roux-en-Y gastric gypass (RYGB) has been performed with low mortality rates and demonstrated high efficiency, especially if the impact on weight loss and comorbidity control is taken into account^1^. RYGB can be considered an incretin and satiating surgery, due to the combination of the restrictive and disabsorptive components, resulting in changes in the hormonal mechanisms involved in controlling the signaling and sensibility of insulin, promoting the homeostasis of glycemia, resulting mainly in the control of DM2 and dyslipidemia^21,28.^

Such technique promotes long term glucose levels normalization in most of the patients, even before body weight loss. The hypothesis of the increase in the peptide incretin production, similar to glucagon 1 - Glucagon like peptide 1 (GLP - 1) in the distal (lower part) intestine was reported in Dirksen et al.^8^ study. Incretins are peptides originated from the intestinal tract that stimulate insulin production in the pancreas, of which those that are part of the entero-insular axis stand out: GLP-1; glucose-dependent insulinotropic polypeptide GIP and peptide yy, PYY^27^.

RYBG can lead to a 35-40% loss of body weight and a 50-80% loss of excess body weight. However, the considerable improvement of glucose control frequently occurs on the first days of the postoperative stage, suggesting remission mechanisms or premature improvement of DM2 levels must be independent from weight loss^6.^

Cho^4^, in her review article, showed that the remission mechanism of diabetes following surgical procedures is still complex and comprises many anatomical and physiological alterations. It's highlighted that caloric restriction, improvement of β cells' functioning and sensibility to insulin, changes in gut physiology, metabolism of bile acids and changes in the intestinal microbiota are seen as potential DM2 remission mechanisms after RYBG.

The objective of this study was evaluating the evolution of DM2, HBP and dyslipidemia on patients submitted to RYBG in the late postoperative period.

## METHOD

This research was conducted in Clementino Fraga Filho University Hospital, Rio de Janeiro RJ, Brazil, where social, anthropometric and nutritional information about the patients were obtained, through the analysis of medical records from the Bariatric Surgery Nutrition program. The study was approved by the Research Ethics Committee of the hospital, under protocol 843.153.

The sampling model adopted for the research was the convenience, according to the eligibility criteria proposed by the researcher, with the objective of obtaining a homogeneous sample. Patients aged between 18-65 years-old, who had been submitted to RYBG between the years of 2000 and 2012 were included in the research. From an initial sample of 87 patients, 59 were enrolled.

The anthropometric variables evaluated were height and body weight. The values obtained in the first consultation were considered as preoperative weight and BMI. In the surgery the weight and BMI values used were from the day of the procedure. In the postoperative stage the weight and BMI values used were the last one registered in the medical records. To calculate the BMI, the formula weight/height squared^30^. Body weight and height were measured according to Gibson^11^, with the use of a digital scale of 300 kg maximum capacity, divided by 50 g and a 0.1 cm scale stadiometer. The percentage of excess weight loss (% EWL) was calculated using the formula: %EWL=(preoperative weight - current weight / preoperative weight - ideal weight) X 100. The BMI used to calculate the ideal weight was of 25 kg/m^2^ ^7^.

For laboratory data the following variables were analyzed: total cholesterol, low density lipoprotein (LDLc), high density lipoprotein (HDLc), very low density lipoprotein (VLDLc), triglyceride (TG) e glucose on the pre and postoperative stages. For evaluating the postoperative data, the last values registered in medical records were considered. Blood analysis was performed at the hospital's laboratory.

### Statistical analysis

Data was analyzed as mean and standard deviation. It was used the Kolmogorov-Smirnov method (Dallal-Wilkinson-Lilliefor p-value) for data normality test. The data regarding glycemia and lipemia were analyzed through paired t tests between the pre and postoperative stages. For the data regarding body weight and BMI, the ANOVA Bonferroni post hoc test was used. To study the relationship between the %EWL coefficients and the laboratory variables, the Pearson correlation coefficient was used, with a 5% significance level. It was considered r>0.70 a strong correlation, from 0.30 to 0.70, a moderate correlation, and from 0 to 0.30 a weak correlation (Collegari-Jaques 2003). P <0.05 was considered as a significant value. The data were analyzed with the Statistical Package for the Social Sciences (SPSS) 21.0 program for Windows.

## RESULTS

The characteristics of the patients evaluated are illustrated on Table 1. From the total of 59 patients, most were female (86%), aged 43+11 and a little more than half of them graduated from high school. Regarding the obesity history, in this same table it's possible to see the problem started in adulthood for a little more than half of the patients and most of them (88%) had records of obesity in the family, with first-degree relatives.

**TABLE 1:** Population characteristics

Sex	% (n)
Female	86 % (38)
Male	14 % (21)
Marital Status	% (n)
Married	56% (33)
Single	39% (23)
Divorced	3% (2)
Widowed	2% (1)
Education	% (n)
Illiterate	3% (1)
Middle school	37% (22)
High school	52% (31)
Higher education/College	8% (5)
Beginning of obesity	% (n)
Childhood	25% (15)
Adolescence	20% (12)
Adulthood	53% (31)
Not informed	2% (1)
Family history of obesity	% (n)
Yes	88 % (52)
No	7% (4)
Not informed	5% (3)
Smoker	% (n)
Yes	12% (7)
No	76% (45)
Not informed	12% (7)

%=percentage of patients; n= number of patients

The average postoperative period was of 7±3 years. Table 2 displays the values of glucose serum concentration, TG, total cholesterol and fractions. It was noticed a decrease of glucose, total cholesterol, LDLc, VLDLc and TG and increase of HDLc levels in the postoperative stage in comparison to the preoperative one, with confidence level of 95%.

**TABLE 2:** Assessment of glycemia and lipemia pre and post operatively

Variables	Preoperative	Postoperative	p
Glucose (mg/dl)	101.00±26.99	89.11±15,19	0.014
Cholesterol total (mg/dl)	179.00±37.95	167.48±28.50	0.016
LDLc (mg/dl)	104.30±33.12	91.46±24.58	0.016
HDLc (mg/dl)	43.53±8.23	57.90±15.60	< 0.001
VLDLc (mg/dl)	25.40±11.12	15.68±7.40	< 0.001
TG (mg/dl)	143.35±86.35	82.45±37.39	< 0.001

LDLc=low density lipoprotein cholesterol; HDLc=high-density lipoprotein cholesterol; VLDLc=lipoprotein very low density cholesterol; TG=triglycerides; p= paired t-test with significance level 0.05

**FIGURE 1:**
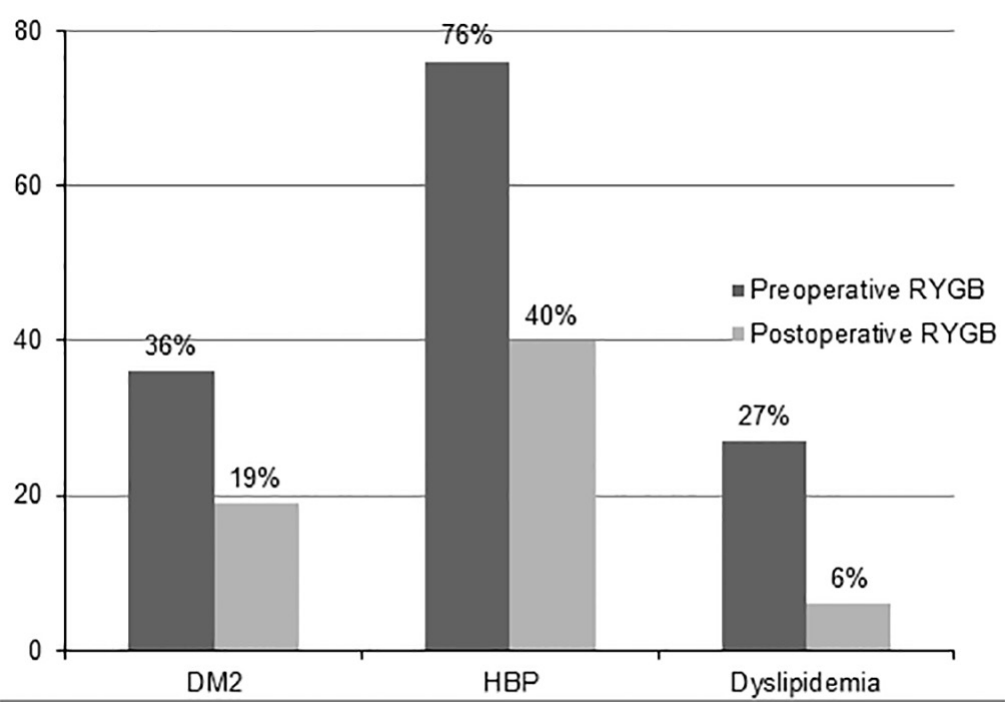
Prevalence of comorbidities in the preoperative and postoperative periods

The most part of subjects (76%) of the present study presented HBP in the preoperative stage; however, 60% had normal blood pressure without need for drug use during the postoperative stage. Regarding DM2 and dyslipidemia, most of the patients were in remission in the postoperative moment, 81% and 94%, respectively. From patient who still present HBP in the postoperative moment, 40% were using one or more types of HBP drugs (Figure 1).

**FIGURE 2:**
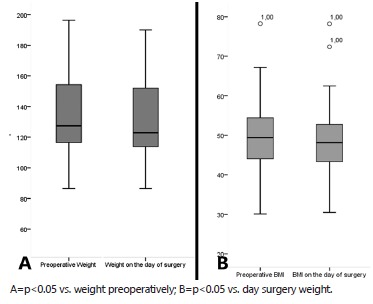
Weight rating (A) and BMI (B) before the day of surgery and after the procedure.

Regarding body weight evolution, BMI is shown in Figure 2. It was verified the reduction of body mass and BMI in the postoperative stage (91.04 kg and 33.79 kg/m^2^) when compared with values obtained in the preoperative stage (133.06 kg and 49.74 kg/m^2^) and the day of the surgery (131.68 kg and 49.21 kg/m^2^). There was not any difference in weight and BMI in the preoperative moment and weight in the surgery. The patients presented %EWL of 65.7 after the surgery.

There was no correlation between the %EWL and glucose (r= -0.206; p=0.185), LDL (r= -0.083; p=0.596), HDL (r= -0.263; p=0.092), VLDL (r= -0.008; p=0.964), total cholesterol (r= 0.098; p=0.516) e TG (r= 0.197; p=0.206).

## DISCUSSION

Over the last decades, the prevalence of obesity has remarkably increased in the Brazilian population. Individuals with severe obesity deal, most of the times, with comorbidities. Because of this context, various surgical procedures have been proposed for treating and controlling obesity and its comorbidities. Indication of surgical intervention has been growing and it is based on a broad analysis of multiple aspects of the patient. Then, changes in lifestyle, including diets, physical activity and behavioral therapy are usually suggested as a way of complementing the multidisciplinary treatment of a multifactorial disease. 

According to data from the latest research on family budget survey (2008-2009)^13^ and Risk Factors Surveillance for Chronic Diseases and Protection through telephone^2^, the increase in the occurrence of obesity happens with both genders; however, throughout the years, with more recurrence for women. Studies from Lehmann et al.^17^ and Rangel et al.^23^ also found prevalence of obesity in women, 75% and 76.6%, respectively. Regarding the age group of those submitted to surgery, Quadros et al.^22^ reported a 40 years old average, supported by a systematic review by Buchwald et al.^3^, in 2004, when 134 studies were analyzed, with a total of 19.338 patients, of whom 72.6% were women, with average age of 39 years old. In the present study, 86% of the patients were women, with average age of 43 years old.

In this research, it was observed that 88% of patients had cases of obesity in family, in which either the father or mother, or both, were obese. It's estimated that between 40-70% of phenotype variation associated to obesity is hereditary. In this regard, genes interfere in the maintenance of body weight and fat over time, through the participation in controlling efferent fibers (leptin, nutrients, synapses), central mechanisms (hypothalamic neurotransmitter) and afferent nerve fibers (insulins, catecholamines, autonomic nervous system)^26^. Moreover, obesity cases in multiple members of the same family confirm the role of genetic heritage in the occurrence of obesity. The probability of obesity in children when the parents are obese was estimated in 50-80%^19^.

The presence of DM2, HBP and dyslipidemia, common comorbidities in patients with severe obesity, was of 36%, 76% and 27%, respectively, before the surgery. This data differs, mainly for HBP, from that found in the meta-analysis performed by Buckwald et al.^3^, which pointed out prevalence of DM2 in 15.3%, HBP in 35.4% and dylipidemia in 35.6% of the patients. On the other hand, Gigante et al.^12^, found prevalence of HBP in 54.4% of their population. According to the VI Brazilian Hypertension Guidelines^29^, the prevalence of HBP is very common and affects one in every four adult individuals in Brazil, regardless of body weight, given that weight excess can aggravate it. In this regard, it's possible to observe that 40% of patients still had the disease in the postoperative stage and were using one or more types of medication.

Authors have noted reversal of DM2 right after RYBG, even before significant weight loss^15^ ^,^ ^16^. The mechanisms by which improvement of DM2 occurs are explained by the presence of the incretins and peptides produced in the small intestine. These substances, represented by GIP, secreted by K cells from the proximal small intestine, and GLP-1, secreted by L cells from the distal small intestine, have its secretion stimulated by the contact with the bolus and, once produced, act upon the pancreas stimulating insulin secretion. Therefore, RYBG, when promoting anatomical approximation between the stomach and the ileus, allows earlier contact between food and distal small intestine, resulting in incretin effect increased of six times more, justifying the improvement and even reversal of DM2, regardless of weight loss^16^ ^,^ ^24^ ^,^ ^25^.

Studies verified reduction of total cholesterol, TG and increase of HDLc after bariatric surgery, and these findings persisted for a long period of time in those patients that maintained weight loss^14^. Jamal et al.^14^ noticed that the improvement of the general lipid profile continued during the six years monitoring after the surgery.

The improvement or reversal of comorbidities after this surgery is well documented in literature with reduction of blood pressure, 80.3% reduction of DM2 and 94% improvement of dyslipidemia^3^. Filho et al.^10^ also reported reduction of glycemic and lipemic in their populations. Similar data was observed in our population, with improvement of HBP, DM2 and dyslipidemia in 60%, 81% and 94% of the patients, respectively.

Studies demonstrate that the 5-10% decrease of total body weight is capable of promoting significant clinical benefits, such as reduction of blood pressure and glycemic concentration^18^. According to Deitel and Greenstein^7^, surgical treatment success occurs when there is body weight excess loss of at least 50%. Although there's controversy surrounding the use of %EWL as a tool for expressing data, it's has been widely regarded as a standard analysis of the surgery's results.

In this population, there were reductions of body weight and BMI when compared to values obtained in the preoperative stage, and according to %EWL, the patients successfully undergo surgical treatment, in the long-term, with a 65.7% result. However, it was not possible to conclude %EWL influenced the improvement of comorbidities, due to absence of correlation.

This study presents limitations due to the difficulty in reaching for a more representative sample of the population, considering the high rates of abandonment of the treatment and the fact it's an institutionalized population. There's also a need for more studies presenting results in the course equal to or higher than five years. Despite the high dropout rates of treatment, it was observed that the population studied reached success in weight loss, even after an average postoperative time of 7±3 years, in addition to DM2 and dyslipidemia reversal and improvement of blood pressure in most of the patients during the postoperative stage. The findings of this study show not only the importance of surgical intervention in severe obesity patients in improving the comorbidities, but also in the maintenance of weight loss over time, in the long-term, being then important for reducing mortality and for the improvement of quality of life.

## CONCLUSION

Roux-en-Y gastric bypass has proven itself to be an effective long term procedure, promoting weight loss, remission of DM2 and dyslipidemia.

## References

[1] Beleli CAV, Camargo MA, Scopin DR (2011). Fatores preditivos na perda ponderal de pacientes submetidos ao Bypass
Gástrico em Y de Roux. BMI bariátrica e metabólica ibero-americano.

[2] Brasil (2010). Ministério da Saúde. Secretaria de Vigilância em Saúde. Secretaria de Gestão
Estratégica e Participativa. Vigitel Brasil 2009: vigilância de fatores de risco e
proteção para doenças crônicas por inquérito telefônico.

[3] Buchwald H, Aviador Y, Braunwald E, Jensen MD, Pories W, Fahrbach K (2004). Bariatric surgery A systemic review and meta-analysis. JAMA.

[4] Cho YM (2014). A Gut feeling to cure diabetes potential mechanisms of diabetes
remission after bariatric surgery. Diabetes Metab J.

[5] Collegari-Jacques SM, Grattapaglia D, Salzano FM, Salamoni SP, Crossetti SG, Ferreira ME (2003). Historical Genetics spatiotemporal analysis of the formation of the
Brazilian population. American Journal of Human Biology.

[6] Cummings DE, Overduin J, Foster-Schubert KE (2004). Gastric by-pass for obesity mechanisms of weight loss and diabetes
resolution. J Clin Endocrinol Metab.

[7] Deitel M, Greenstein RJ (2003). Recommendations for reporting weight loss. Obes Surg.

[8] Dirksen C, Jorgensen NB, Bojsen-Moller KN, Jacobsen SH, Hansen DL, Worm D (2012). Mechanisms of improved glycaemic control after RouxenY gastric
bypass. Diabetologia,.

[9] Ferraz EM, Arruda PCL, Bacelar TS, Ferraz AAB, Albuquerque AC, Leão CS (2003). Tratamento cirúrgico da obesidade mórbida. Rev Col Bras Cir.

[10] R D, David IMB, Pacini JF, Miksche LC, Campos EMB, Moraes JC (2009). Avaliação de níveis lipêmicos e glicêmicos pré e pós-cirurgia
bariátrica. Rev Bras Clin Med.

[11] Gibson DJ, Harnett DC, Merril LS (1990). Fire temperature heterogeneity in constrasting fire prone habitats
Kansas tallgrass prairie and Florida sandill. Bulletin of the Torrey Botanical Club.

[12] Gigante DP, MOURA EC, SARDINHA LMV (2009). Prevalence of overweight and obesity and associated factors, Brazil,
2006 Rev. Saúde Pública.

[13] Instituto Brasileiro de Geografia e estatística (2010). Pesquisa de Orçamentos Familiares 2008-2009: Antropometria e estado
nutricional de crianças adolescentes e adultos no Brasil.

[14] Jamal M, Wegner R, Heitshusen D, Liao J, Samuel I (2011). Resolution of hyperlipidemia follows surgical weight loss in patients
undergoing Roux-en-Y gastric bypass surgery a 6-year analysis of
data. Surg Obes Relat Dis.

[15] M FC, S WS, S N, Ferreira PAM, Araújo GF, Mandarino NR (2009). Efeito da perda ponderal induzida pela cirurgia bariátrica sobre a
prevalência de síndrome metabólica. Arq Bras Cardiol.

[16] Laferrére B, Teixeira J, McGinty J, Tran H, Egger JR, Colarusso A (2008). Effect of weight loss by gastric bypass surgery versus hypocaloric
diet on glucose and incretin levels in patients with type 2
diabetes. J Clin Endocrinol Metab.

[17] Lehmann AL, Valezi AC, Brito EM, Marson AC, Souza JCL. (2006). Correlação entre hipomotilidade da vesícula biliar e desenvolvimento
de colecistolitíase após operação bariátrica. Rev Col Bras Cir.

[18] Lottenberg AMP (2006). Tratamento dietético da obesidade. Einstein.

[19] Macho-Azcarate T, Marti A, Martinez JA, Ibãnez J (2002). Gln27Glu polymorphism in the beta2 adrenergic gene and lipid
metabolism during exercise in obese women. Int J Obes Relat Metab Disord.

[20] Oliveira LF, Tisott CG, Silvano DM, Campos CM, Nascimento RR (2015). Glycemic behavior in 48 hours postoperative period of patients with
type 2 diabetes mellitus and non diabetic submitted to bariatric
surgery. Arq Bras Cir Dig.

[21] Pajecki D, Santo MA, Joaquim HD, Morita F, Riccioppo D, de Cleva R, Cecconello I (2015). Bariatric surgery in the elderly results of a mean follow-up of five
years. Arq Bras Cir Dig.

[22] Quadros MRR, Savaris AL, Ferreira MV (2007). Intolerância alimentar no pós-operatório de pacientes submetidos à
cirurgia bariátrica. Rev Bras Nut Clin.

[23] Rangel LOB, Faria VSP, Magalhães EA, Araújo ACT, Barros EMRD (2007). Perfil de saúde e nutricional de pacientes portadores de obesidade
mórbida candidatos à cirurgia barirátrica. Rev Bras Nut Clin.

[24] Silva PT, Patias LD, Alvarez Gda C, Kirsten VR, Colpo E, Moraes CM (2015). Profile of patients who seek the bariatric surgery. Arq Bras Cir Dig.

[25] Silva-Neto EF, Vázquez CM, Soares FM, Silva DG, Souza MF, Barbosa KB (2014). Bariatric surgery reverses metabolic risk in patients treated in
outpatient level. Arq Bras Cir Dig.

[26] Snyder EE (2004). The human obesity gene map: the 2003 update. Obes Res.

[27] Thaler JP, Cummings DE (2009). Minireview: Hormonal and metabolic mechanismsof diabetes remissiton after
gastrointestinal surgery.

[28] Varaschimi M, Nassif PAN, Moreira LB, Nascimento MM, Vieira GMN, Garcia RF (2012). Alterações dos parâmetros clínicos e laboratoriais em pacientes obesos
com diabetes melito tipo 2 submetidos à derivação gastrojejunal em y de Roux sem
anel. Rev Col Bras Cir.

[29] (2010). VI Diretrizes Brasileiras de Hipertensão. Arq Bras Cardiol.

[30] World Health Organization (2000). Obesity: preventing and managing the global epidemic. Report of a WHO
Consultation.

